# Intranasal post-cardiac arrest treatment with orexin-A facilitates arousal from coma and ameliorates neuroinflammation

**DOI:** 10.1371/journal.pone.0182707

**Published:** 2017-09-28

**Authors:** Hiren R. Modi, Qihong Wang, Sahithi GD, David Sherman, Elliot Greenwald, Alena V. Savonenko, Romergryko G. Geocadin, Nitish V. Thakor

**Affiliations:** 1 Department of Biomedical Engineering, The Johns Hopkins University School of Medicine, Baltimore, Maryland, United States of America; 2 Departments of Pathology and Neurology, The Johns Hopkins University School of Medicine, Baltimore, Maryland, United States of America; 3 Neurology and Anesthesiology/Critical Care Medicine, The Johns Hopkins University School of Medicine, Baltimore, Maryland, United States of America; Fraunhofer Research Institution of Marine Biotechnology, GERMANY

## Abstract

Cardiac arrest (CA) entails significant risks of coma resulting in poor neurological and behavioral outcomes after resuscitation. Significant subsequent morbidity and mortality in post-CA patients are largely due to the cerebral and cardiac dysfunction that accompanies prolonged whole-body ischemia post-CA syndrome (PCAS). PCAS results in strong inflammatory responses including neuroinflammation response leading to poor outcome. Currently, there are no proven neuroprotective therapies to improve post-CA outcomes apart from therapeutic hypothermia. Furthermore, there are no acceptable approaches to promote cortical or cognitive arousal following successful return of spontaneous circulation (ROSC). Hypothalamic orexinergic pathway is responsible for arousal and it is negatively affected by neuroinflammation. However, whether activation of the orexinergic pathway can curtail neuroinflammation is unknown. We hypothesize that targeting the orexinergic pathway via intranasal orexin-A (ORXA) treatment will enhance arousal from coma and decrease the production of proinflammatory cytokines resulting in improved functional outcome after resuscitation. We used a highly validated CA rat model to determine the effects of intranasal ORXA treatment 30-minute post resuscitation. At 4hrs post-CA, the mRNA levels of proinflammatory markers (IL1β, iNOS, TNF-α, GFAP, CD11b) and orexin receptors (ORX1R and ORX2R) were examined in different brain regions. CA dramatically increased proinflammatory markers in all brain regions particularly in the prefrontal cortex, hippocampus and hypothalamus. Post-CA intranasal ORXA treatment significantly ameliorated the CA-induced neuroinflammatory markers in the hypothalamus. ORXA administration increased production of orexin receptors (ORX1R and ORX2R) particularly in hypothalamus. In addition, ORXA also resulted in early arousal as measured by quantitative electroencephalogram (EEG) markers, and recovery of the associated behavioral neurologic deficit scale score (NDS). Our results indicate that intranasal delivery of ORXA post-CA has an anti-inflammatory effect and accelerates cortical EEG and behavioral recovery. Beneficial outcomes from intranasal ORXA treatment lay the groundwork for therapeutic clinical approach to treating post-CA coma.

## Introduction

Cardiac arrest (CA) entails significant risk of coma or disorders of consciousness resulting in poor neurological outcome after resuscitation [1,2]. Therapeutic hypothermia has been shown to provide some benefits in post-CA survival and improved neurological outcomes in clinics and preclinical studies [3–5][6]. However, recent findings from Targeted Temperature Management (TTM) trial contest the view that hypothermia (33°C) would offer an advantage over 36°C [6]. While deep hypothermia may still hold promise of beneficial effects, there is an unmet need to explore alternative approaches to facilitate arousal and neuroprotection [7,8]. The orexinergic pathway has emerged as an important part of the brain’s arousal system [7,9]. The brain stem and diencephalic nuclei modulated by the orexinergic system regulate not only arousal but also basic physiologic functions [10,11]. It originates in the hypothalamus and delivers orexin to different brain regions. Orexin-A (ORXA) is a neuropeptide that is responsible for maintaining wakefulness and is closely associated with other neurotransmitter pathways [9,12,13].

Global cerebral ischemia induced by CA and followed by reperfusion triggers a multitude of processes that ultimately result in neuroinflammatory responses with activation of glial cells, release of proinflammatory cytokines, and delayed neuronal death [14–17]. Levels of IL-6 and TNF-α were shown to be strongly associated with severity of post-CA syndrome (PCAS) [18], mortality rate, and neurologic outcomes [19–22]. Importantly, the systemic inflammatory response after CA was not modified by therapeutic hypothermia at 33°C or 36°C. Despite the pathophysiologic significance of CA-induced inflammation in general and neuroinflammation in particular [18,20–23], the potential of anti-inflammation therapeutic approaches in limiting consequences of CA has not been adequately studied. Recent studies have suggested that ORXA, in addition to its well-established role in promoting arousal [7,9], may also exhibit anti-inflammation properties [24–27]. Combination of such effects makes ORXA a particular attractive candidate for the treatment of CA-associated coma and neuroinflammation.

Our previous work explored ORXA as a drug that may promote arousal in an experimental setting of CA-induced coma [12]. A single intracerebroventricular (icv) injection of ORXA resulted in an improvement in the EEG-based measure of arousal at 4 hrs post CA. However, in the previous work we did not test whether post-CA ORXA treatment could result in any detectable changes in cytokine production in the brain that would support the idea of its anti-inflammatory properties. In the present study, we have changed the route of administration to increase a potential translational value of the post-CA ORXA treatment. We used an intranasal delivery of ORXA that can be easily implemented in clinical settings. In this proof-of-concept study we have opted to test beneficial effects of ORXA at the time point at which the icv ORXA treatment has already been proven effective (4hrs) [12]. We have accessed ORXA effects on neurological arousal by means of quantitative EEG that in our previous studies has been sensitive to severity of CA-associated injury as well as predictive of behavioral outcomes after hypothermia [28,29] and icv-ORXA treatment[12,30]. The development of our quantitative EEG approaches during the previous studies has suggested that an EEG-derived gamma frequency band might be a particular promising tool for assessing neurological arousal. Behavioral outcomes were analyzed by a neurological deficit score (NDS) [31,32] that is a well-accepted standard to characterize effects of CA [33–35] and has been used in our previous study of post-CA ORXA treatment [12].

In the current study, we have tested the hypothesis that intranasal delivery of ORXA will improve neurological and behavioral outcomes of CA-induced coma. An additional important focus of our study is the exploration of the pathways involved in ORXA action. We hypothesize that ORXA will mitigate the neuroinflammatory response. The goals of our study are to demonstrate the effects of intranasal ORXA treatment on facilitating arousal from coma (as judged by behavioral and cortical EEG outcomes), and, in addition, ameliorate neuroinflammatory responses in a brain region-specific manner. These studies should serve as a proof-of-concept for intranasal delivery of ORXA, and should give an impetus to further clinical development of the treatments that successfully combine pro-arousal and anti-inflammatory effects.

## Materials and methods

### Animal model

#### Subjects and implantation of electrodes

Adult male Wistar rats, (300-350g; Charles River, Wilmington, MA) were utilized for this project. The study was designed as a proof-of-concept experiment for intranasal administration of ORXA. Our previous experiments with icv delivery of ORXA were conducted in males [12], so we have chosen to use animals of the same sex to avoid possible interference of an additional factor (sex) with the effects of intranasal ORXA. All experimental procedures were approved by the Institutional Animal Care and Use Committee at the Johns Hopkins University School of Medicine. The animals were habituated for 1 week before electrode implantation. Electrodes implantation was conducted under 2–3% isoflurane anesthesia delivered via a mask. Rats were implanted with 5 epidural EEG screw electrodes (Plastics One, USA), with two of the electrodes placed at 2mm anterior to Bregma and 2mm to the right and left of Bregma, corresponding to the M1 regions of the frontal cortex. Additional two electrodes were placed at 6mm posterior to Bregma and 4mm to the right and left of Bregma, corresponding to the V1 regions of the occipital cortex. A ground/reference electrode was placed over the cerebellar region (2 mm posterior to Lamda). The electrodes were attached to a pedestal (MS363, Plastics One, USA) and stabilized with denture resin (Lang Dental, USA) to facilitate connection with the neural recording device, an RX5 TDT device (Tucker Davis Technologies, Alachua, FL). The electrodes were implanted 1 week prior to asphyxia-induced CA experiments to allow full recovery from this preliminary preparation [12,30].

#### Asphyxial-CA model

**Fig 1:**
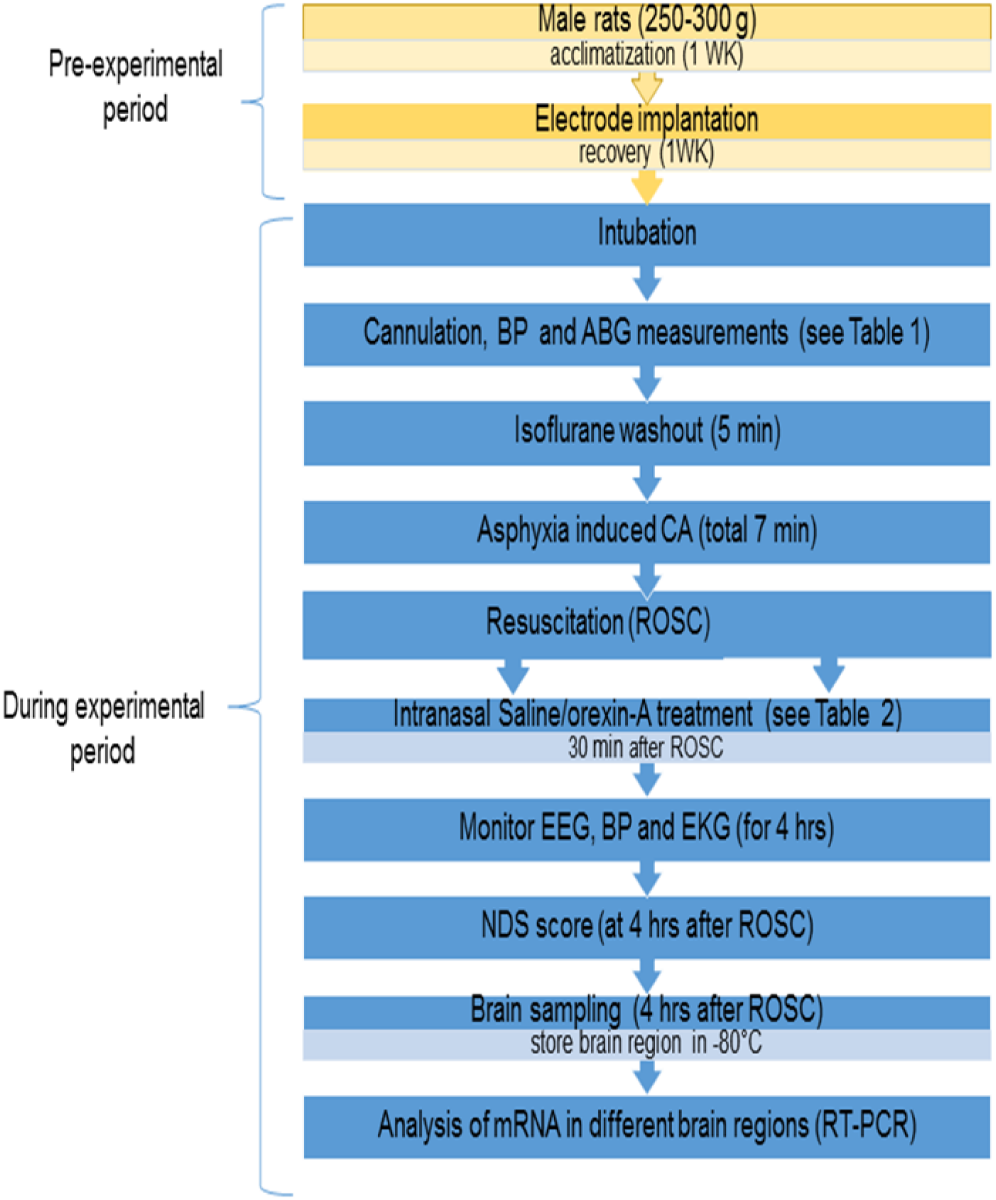
Schematic diagram of experimental design.

One week after electrode implantation, animals were subjected to asphyxia-induced 7 min CA (Fig 1). Based on the prior studies [12,30,36], we have expected that this duration results in an injury that is broad and significant but still results in the survival of the animal post CA, and thus allowing examination and titration of neuroprotective strategies. Our previous study has also demonstrated that CA in this model results in neuronal injury when tested as early as 4hrs post-ROSC [37]. Prior to CA, rats were endotracheally intubated by direct laryngoscopy and mechanically ventilated with 2% isoflurane in 50% O_2_ + 50% N_2_ gas, after which the femoral artery and vein were cannulated with polyethylene 50 tubing catheters to monitor blood pressure (BP) and sample arterial blood gases (ABGs) and to administer intravenous medications. Our CA protocol has been described in detail previously [12,30,36]. Briefly, we carried out 10 minutes of baseline EEG recording under isoflurane. Following baseline recording, anesthesia was washed out for a total 5 minutes, starting with 2 minutes of 100% O_2_ without isoflurane to capture non-anesthetized EEG. Then for 3 minutes, FiO_2_ was decreased to 20% with 80% of N_2_ gas (room air). Rocuronium bromide 2mg/kg IV was administered for muscle paralysis at 2 minutes of washout time. Following 5 minutes of gas washout, global asphyxia was induced by stopping and disconnecting the ventilator and clamping the tracheal tube. Global asphyxia was accompanied by transient hypertension, followed by progressive bradycardia, hypotension and eventual CA (defined by MAP<10 mmHg and cessation of electrical rhythm and non-pulsatile-pressure wave) are observed.

### Resuscitation

After the predetermined CA period (7 min), cardiopulmonary resuscitation (CPR) was initiated by unclamping the tracheal tube, restarting mechanical ventilation with 100% O_2_, administering epinephrine (5 μg/Kg, i.v.), and applying sternal chest compressions with two fingers (~200 compressions/min, to generate mean arterial pressure (MAP) >50 mmHg) and NaHCO3 (1mmol/Kg, i.v.) to normalize arterial pH. When the aforementioned measures were achieved, the resulting time was noted as a return of spontaneous circulation (ROSC). ABGs were sampled 15 minutes after ROSC. Anesthesia was not provided post-resuscitation in order to minimize drug effects on recorded neural (EEG) signals and to facilitate the return of arousal. Following successful resuscitation, the animal was hyperventilated (TV 10 ml/Kg, RR 65/min and PEEP 3 cmH_2_O) for 10 min. Subsequent ventilator changes were performed according to the ABG. RR was adjusted to 50/min [12,36]. Rectal temperature was maintained at 37.0°C using a warming pad and lamp. Once a rat achieved spontaneous respirations, it was extubated, and vascular catheters were removed, sutures were administered to the femoral region.

### Treatment with Orexin-A (ORXA)

At 30 minutes post-ROSC, the rats were randomized to receive saline (vehicle), low (10μM) ORXA or high dose (50 μM) of ORXA intranasally. Each animal received 10 μl x 3 in each nostril for a 30 second interval (60 μl total). The low dose ORXA group received 600 nmoles (10μM/L = 10nmoles/1 μL; 10 nmoles X 60 μl = 600 nmoles) and high dose (50 μM) ORXA group received 3000 nmoles total, respectively.

### Behavioral testing

The Neurologic Deficit Scale score (NDS score), which was previously developed and validated extensively [31,32,37]ranges from 0–80 and serves as a surrogate quantitative rodent neurodeficit and coma score, analogous to human coma scores. The NDS was previously used to quantitate rodent coma and arousal levels [36,38]. The NDS assesses arousal, cranial nerve reflexes, and motor behavior. See S1 Table for details of its components. The NDS was determined at 4 hours post-ROSC. Trained personnel who were blinded to the vehicle and treatment groups conducted NDS examinations (S1 Table).

### EEG recording and analysis

Before performing CA, baseline EEG (15 minutes) was recorded. The signals were digitalized using the data acquisition package CODAS (DATAQ Instruments INC., Akron OH). EEG was monitored for 4 hours post-CA continuously. The EEG was down sampled from 12,200 samples per second to 122. EEG gamma band (γ = 30-50Hz) energy was calculated using the Teager Energy Operator [39]. Additionally, delta (1–3 Hz), theta (4–7 Hz), alpha (8–12 Hz) and beta (21–30 Hz) energy bands were also calculated for every sample. The Gamma Fraction was obtained by dividing the gamma band energy by the total power under 50Hz. These measurements were then averaged over one minute. Gamma band EEG was previously validated as a measure of arousal from coma [30,36,38,40].

### Brain samples collections

Four hours after CA and resuscitation, the animal was anesthetized with isoflurane and then decapitated. The brain was rapidly excised and the frontal cortex (PFC), caudal cortex, hippocampus, striatum, hypothalamus, medulla, rest of the brain (ROB), and cerebellum were dissected. Each brain region was frozen in 2-methylbutane at −50°C, and stored at −80°C until use.

### Total RNA isolation and real time RT-PCR

Our choice of proinflammatory markers was mainly based on two criteria: whether the markers are activated in the early phase of inflammation and how well they are characterized in the models of ischemia. By these criteria, IL-1β, TNFα, and iNOS are all involved in an acute phase of inflammation and are widely used markers of neuroinflammation induced by ischemia [14,41]. GFAP was used as a marker of astrocytes and increases in GFAP transcription could be considered as a marker of initiation of astrogliosis. CD11b has been chosen as a widely used marker of microglial activation in the brain in settings of proinflammatory responses. Total RNA was isolated from different brain structures using an RNeasy lipid tissue mini kit (Qiagen, Valencia, CA). Briefly, the tissue was homogenized in Qiagen lysis solution and total RNA was isolated by phenol-chloroform extraction. Complementary DNA was prepared from total RNA using a high-capacity cDNA Archive kit (Applied Biosystems, Foster City, CA). mRNA levels (IL1β, iNOS, CD11b, TNF-α, GFAP, ORX1 R, ORX2 R) were measured by quantitative RT-PCR, using an ABI PRISM 7000 sequence detection system (Applied Biosystems). Specific primers and probes for these markers (S2 Table), purchased from TaqManR gene expression assays (Applied Biosystems), consisted of a 20X mix of unlabeled PCR primers and Taqman minor groove binder (MGB) probe (FAM dye-labeled). The fold-change in gene expression was determined by the ΔΔCT method [42]. Data are expressed as the relative levels of the target gene in the CA rat normalized to the endogenous control (GAPDH) and relative to the control (sham control). All experiments were carried out in duplicate with 10 sham controls, 6 brain samples for CA+saline and 6 brain samples from CA+ORXA treatment rats. Data are expressed as relative fold change in gene expression.

### Statistics

Data are presented as mean ± SEM. When three groups were compared (sham control, saline treated CA and ORX treated CA), statistical significance was determined using main effect or mixed design ANOVAs followed by post-hoc tests when appropriate for comparisons between particular set of means. Statistical significance was set at p ≤0.05. P levels for multiple comparisons were modified by Bonferroni correction. Details of statistical analyses for behavioral and EEG data are presented in S3, S4 and S5 Tables, respectively. Results of statistical analyses for mRNA levels of cytokines and ORX receptor levels in different brain structures are presented in S6–S9 Tables.

## Results

### Baseline characteristics of ORXA treated and control saline animals

We obtained baseline characteristics of body weight, blood gases and hemoglobin levels for all rats immediately prior to CA (Table 1). No statistical differences were observed for these parameters between groups, confirming successful randomization into the treatment groups (Table 1). Baseline hemodynamic parameters, including heart rate (HR) and BP measurements as obtained by arterial line measurements prior to ORXA or saline treatment did not show any significant differences as well (Table 2).

**Table 1:** Baseline (pre-cardiac arrest) characteristics of Orexin-A and control groups. No significant differences in baseline characteristics between animals in CA+Saline (n = 6) and CA+Orexin-A (50 μM) (n = 6).

Parameters	CA+Saline	CA+Orexin-A
	(n = 6)	(n = 6)
Weight(g)	367.4±7.59	365.12±3.11
pH	7.44±0.12	7.37±0.14
pCO2 (mmHg)	40.05±1.74	39.12±2.39
pO2 (mmHg)	162.2±8.18	177.66±16.43
HCO3 (mmol/L)	27.52±0.57	29.33±0.46
Na (mmol/L)	136.93±0.43	136.00±0.40
Hemoglobin (mmol/L)	12.01±0.25	12.33±0.09
Time to CA(s)	243.33±4.81	241.5±8.54
Time to ROSC(sec)*	96.33±13.14	102.5±6.76

Group means ± SEM are shown.

*ROSC indicates return of spontaneous circulation.

**Table 2:** Hemodynamic parameters at 30 minutes post- ROSC (before drug) and 30 minute post Orexin-A treatment. No significant differences were observed before treatments. The effects of ORXA (50 μM) or Saline were measured 30 min after the treatment.

GROUP	30 minutes Post-ROSC (Pre-Treatment Drug)		30 minutes Post-Drug (Post-Treatment Drug)	
	CA+ Saline (n = 6)	CA+ Orexin-A (n = 6)	CA+ Saline (n = 6)	CA+ Orexin-A (n = 6)
SBP (mmHg)	130.93±6.14	143.66±10.48	106.32±7.95^#^	130.51±6.74
DBP (mmHg)	86.90±4.04	88.32±6.79	74.25±6.38^#^	86.73±5.80
MAP (mmHg)	101.64±4.67	106.77±7.93	86.49±6.77*^#^	106.63±6.37
Pulse (bpm)	380.23±6.60	373.82±23.07	340.06±11.52^#^	336.42±10.86^#^

Group means ± SEM are shown. ROSC indicates return of spontaneous circulation; SBP, systolic blood pressure; DBP, diastolic blood pressure; and MAP, mean arterial pressure; bpm, beats per minute.

Two-way mixed design ANOVAs (Group x Time point) were used for statistical analyses. An asterisk (*) indicates a significant between-group difference at the post-drug period, p<0.05; pound (#) indicate significant effects of Time point (Post-ROSC vs Post-Drug) in the same group of animals, p <0.05 (Neuman-Keuls post-hoc test applied to a significant ANOVA main effect or interaction).

### CA induced coma

As illustrated in Table 1, times to enter CA or return of spontaneous circulation (ROSC) were statistically equivalent between groups. In our CA model (7 min-long CA), the failure to achieve ROSC (failure of resuscitation) is about 10%. After this protocol of CA, all animals remained in coma. They usually started regaining alertness around 4 hrs post-CA.

### Hemodynamic response to ORXA treatment

Hemodynamic measurements in the saline-treated rats revealed significant decreases in heart rate and blood pressure between 30 and 60 min after ROSC (Fig 1; Table 2). In contrast to the saline group, the ORXA-treated rats showed more stable parameters of hemodynamics during this time period (Table 2). It is noteworthy that this effect of ORXA treatment was limited to only blood pressure but not heart rate (Table 2).

### Orexin-A improves behavioral arousal (NDS)

**Fig 2:**
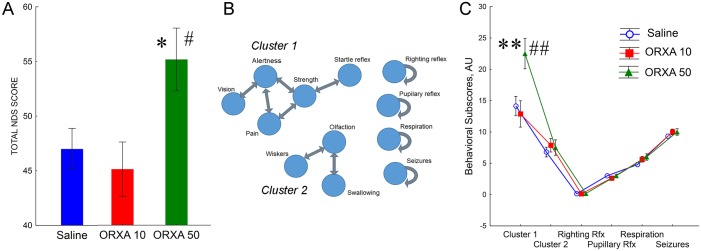
Intranasal orexin-A treatment improves behavioral outcomes after CA. (A) Neuro Deficit score (NDS) was tested at 4 hrs after ROSC for control (CA+ saline) (n = 6), CA+10μM orexin-A (n = 6), and CA+ 50μM orexin-A (n = 6) treatment groups. (B) Schematic representation of correlations between individual NDS subscores. See S3 Table for correlation coefficients. (C) Subscores of Cluster 1 were improved after treatment with 50 μM ORXA. Values are reported as mean± SEM. Single and double asterisks indicate significant differences from Saline group as a result of LSD post-hoc test with p levels less than 0.025 or 0.0001, respectively. Single and double pound signs indicate significant differences from ORXA-10 group as a result of Bonferroni post-hoc test with p levels less than 0.025 or 0.0001, respectively. See S2 Table for the results of appropriate ANOVAs.

Neurologic testing of rats was conducted at 4 hrs post-ROSC in both saline and ORXA treated groups (Fig 2). To analyze a dose-response of behavioral effects of ORXA, two experimental groups were included (ORXA-10μM and ORXA-50μM). Total NDS score consists of sum from 18 different tests with the maximum of 80 points (best performance) and the minimum 0 points (worst performance) (S1 Table). At 4 hrs after ROSC, comparison of total NDS scores between the saline, ORXA-10 and ORXA-50 groups revealed better performance in the rats treated with 50μM of ORXA as compared with two other groups (Fig 2A, S2 Table). Lower dose of ORXA (10μM) failed to show efficacy as there were no significant differences between the saline and ORXA-10 groups. Since NDS testing is designed to reflect a full range of behavioral repertoire from arousal to coma, not all categories of behavioral responses are sensitive at particular states of arousal. In the next set of analyses, we removed all behavioral responses that showed no variability due to “floor/ceiling” effects. This yielded a set of 12 variables. To reveal possible correlations within this set of variables the data were submitted to Spearman Rank Order correlations (S3 Table). This analysis revealed two clusters of correlated variables (Fig 2B). Cluster 1 included NDS subscores of alertness, vision, pain reactivity, strength and acoustic startle reaction, while Cluster 2 reflected olfaction, swallowing, and reaction to whisker stimulation (Fig 2B). The pupillary and righting reflexes as well as indexes of respiration and seizures were not correlated to any other subscores (Fig 2B). Analysis of the effect of ORXA treatments on NDS subscores revealed a significant improvement in behavioral outcomes due to an increase in subscores of Cluster1 after treatment with 50μM ORXA (Fig 2C, S2 Table). There were no significant improvements for the 10μM ORXA treatment group.

Based on behavior outcome results (NDS results) post-CA, we took this study forward using 50μM ORXA treatment to evaluate neurophysiological arousal and its anti-inflammatory effect in the brain.

### Orexin-A promotes arousal after CA/resuscitation as measured by EEG gamma fraction

**Fig 3:**
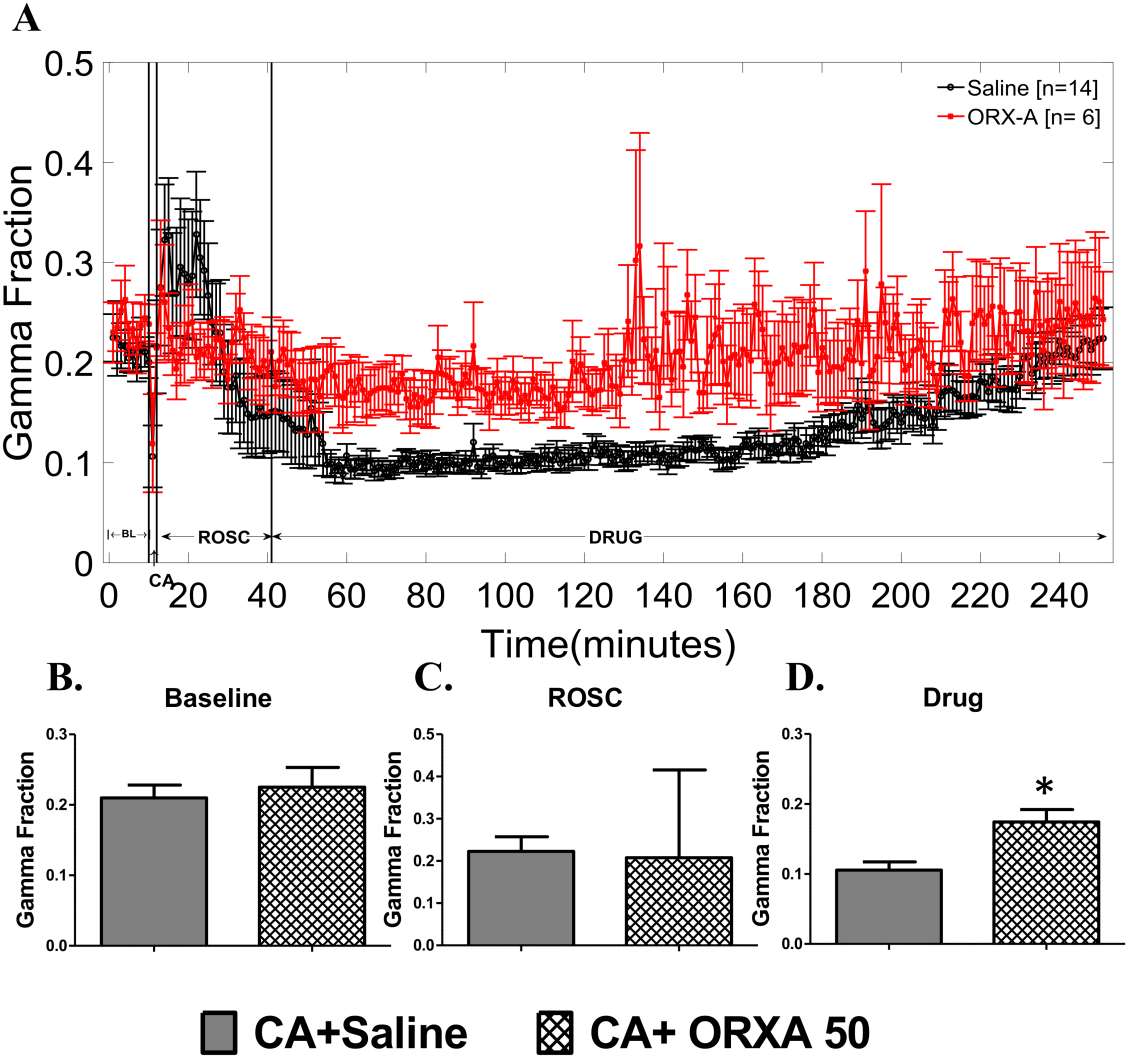
EEG IQ gamma band (30–50 Hz) for the50μM Orexin-A group (red line, n = 6) and the group of control rats treated with saline (black line, n = 6). (A) Sub-band EEG gamma power tracing shows that orexin-A leads to improved EEG IQ shortly after intranasal administration in gamma band EEG IQ levels. (B-D) EEG gamma power averaged for periods of baseline recording (B), ROSC (C), and post-treatment (Saline/ ORXA) (D). Data are presented in mean± SEM. BL indicates baseline (pre-anesthesia); CA: asphyxial cardiac arrest; Drug: represents the period after saline or orexin treatment. Asterisk indicates significant between-group differences for the post-drug period, p<0.021. Results of statistical analyses are presented in S4 Table.

Next, we investigated the effect of intranasal 50μM ORXA on EEG gamma band power (30–50 Hz), a measure of neurophysiologic arousal in the brain. The EEG gamma band has also been shown to be a predictor of good neurological outcome [43]. EEG gamma power was indistinguishable between groups before treatments (Fig 3A–3C, S4 Table) supporting a successful randomization into the groups. After treatments, the ORXA-50 group demonstrated significantly higher EEG gamma power when tested during ~3.5hrs post-treatment period (Fig 3A and 3D, S4 Table). Significant effect of ORXA-50 was revealed mainly due to differences observed from 45 to 165 min post treatment (Bonferroni post-hoc test, p<0.05) (Fig 3A). At later time points the differences between the saline and the ORXA-50 groups disappeared due to recovery of EEG gamma band in saline treated animals. Thus, intranasal treatment with 50μM ORXA improved early recovery of EEG gamma power, particularly in the first 2.5 hrs after the treatment.

### Post-CA Orexin-A treatment primarily increases mRNA levels of ORX type 1 receptor

**Fig 4:**
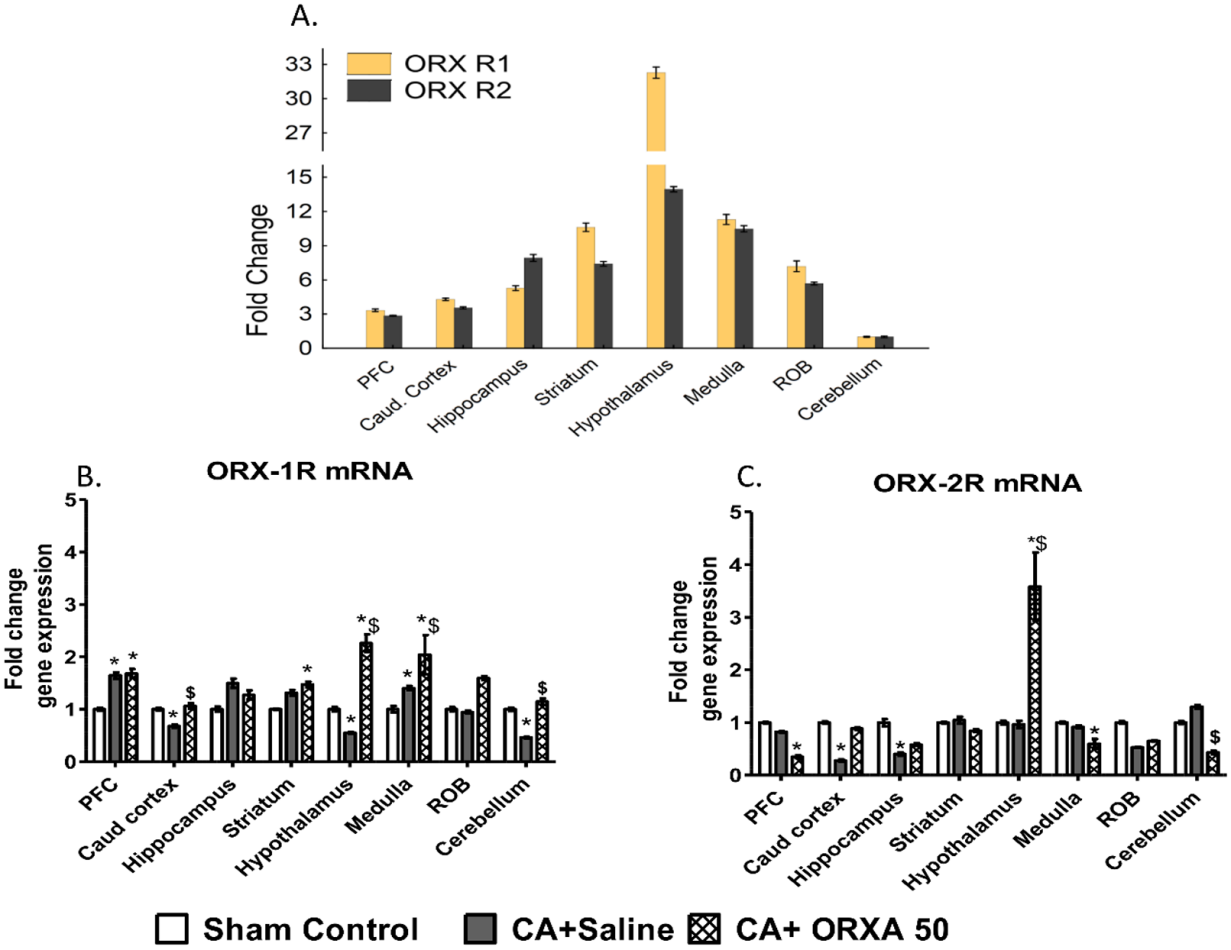
Effects of CA and ORXA treatment (50 μM) on mRNA levels of ORX receptors in different brain structures. (A) mRNA levels of Orexin receptor 1 and 2 in different brain structures as compared to Cerebellum. (B-C) Effects of CA and ORXA treatment on mRNA levels of ORX receptor-1 (B), and ORX receptor-2 (C). Results are expressed relative to the control group. Asterisks and pound sings indicate significant differences from Sham control or CA+Saline group, respectively, as a result of LSD post-hoc tests with p levels corrected for multiple comparisons, p_cor_<0.0021 (see S5 Table for results of ANOVA). Probe sequences for RT-PCR are presented in S2 Table.

There are two types of receptors to orexin, and ORXA neuropeptide can bind and activate both orexin receptor 1 (ORX 1R) and orexin receptor 2 (ORX 2R) [44]. mRNA levels of ORX receptors are characterized by dramatic variability between different brain structures with the maximum levels detected in the hypothalamus for both types of receptors (Fig 4A). CA-induced changes in mRNA levels of ORX 1R were also dependent on a brain structure and varied from activation (PFC, Medulla) to inhibition (Caudal Cortex, Hypothalamus, Cerebellum) (Fig 4B). Predominant effect of post-CA treatment with ORXA was an increase in ORX 1R mRNA levels as compared to control or CA-Saline group (Fig 4B). In contrast to ORX 1R, ORX 2R mRNA levels were mostly decreased as a result of CA and/or ORXA treatment. The only exception was the hypothalamus where, in concert with ORX 1R, mRNA levels of ORX 2R were dramatically increased after the ORXA treatment (Fig 4C). Despite the contrasting character of changes in mRNA levels of ORX 1R and ORX 2R, within each treatment group mRNA for both types of receptors remain correlated (S1 Fig; S6 Table). In summary, effects of post-CA treatment with ORXA predominantly increase mRNA levels of orexin receptor 1 while mRNA levels of orexin receptor 2 remain unchanged or decrease. The only brain structure in which ORXA treatment increase mRNA levels of both types of orexin receptors is the hypothalamus. In addition, analyses of variability in brain structures and treatment groups imply existence of multiple mechanisms subserving structure and state-specific co-regulation of levels of ORX 1R and 2R receptors.

### Post-CA Orexin-A treatment has anti-inflammatory effect in the brain

**Fig 5:**
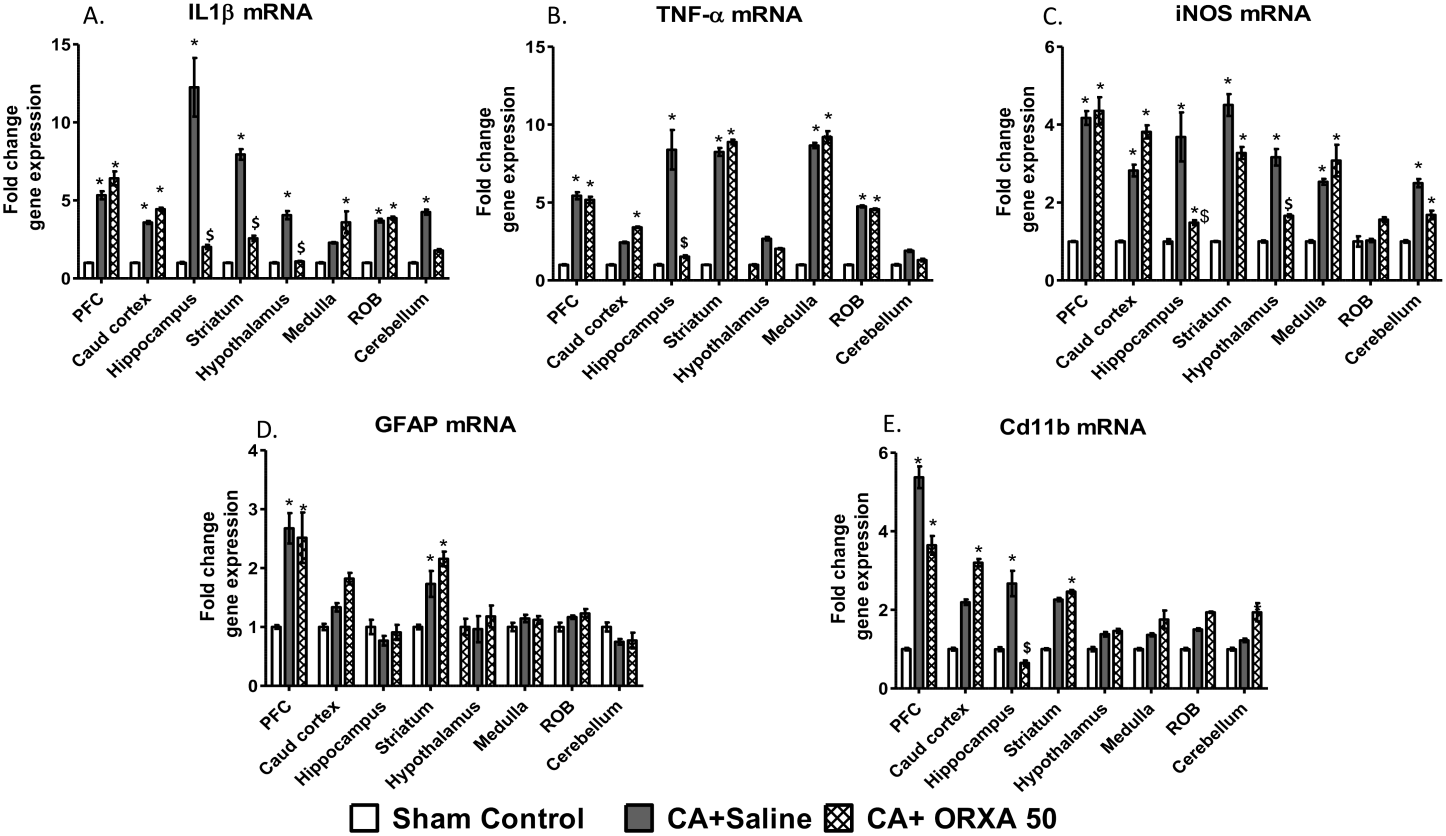
Effects of CA and ORXA treatment (50 μM) on mRNA levels of neuroinflammatory markers. (A) CD11b, (B) iNOS, (C) TNF-α, (D) GFAP, and (E) IL1β. RT-PCR measures were done in brain samples of the prefrontal cortex (PFC), caudal cortex, hippocampus, striatum, hypothalamus, medulla, rest of the brain (ROB), and cerebellum. Asterisks and pound sings indicate significant differences from Sham control or CA+Saline group, respectively, as a result of LSD post-hoc tests with p levels corrected for multiple comparisons, p_cor_<0.0021 (see S7 Table for results of ANOVA). Probe sequences for RT-PCR are presented in S2 Table.

To characterize a neuroinflammatory status after post-CA ORXA treatment, we chose five well-characterized markers of neuroinflammation that represent activation of cytokines (IL1β, TNFα), nitric oxide (iNOS), changes in an astrocytic structural protein (GFAP), and microglial cell surface marker (CD11b). To avoid possible issues with different sensitivity of antibodies in western blot or histochemistry assays, we chose to characterize the neuroinflammatory markers by the RT-PCR approach. IL1β cytokine is an important mediator of the inflammatory response, and is involved in a variety of cellular activities, including cell proliferation, differentiation, and apoptosis [45]. The induction of cyclooxygenase-2 (PTGS2/COX2) by this cytokine in the central nervous system is found to contribute to inflammatory pain hypersensitivity and other neurological disorders [41]. Among proinflammatory molecules, IL1β is considered as an immediate-response protein secreted early by activated microglia and astrocytes [46]. In the Saline treated group, CA dramatically increased IL1β mRNA expression levels within all brain regions as compared to sham operated control. Particularly vigorous responses were observed in the hippocampus, striatum, and prefrontal cortex (PFC) (Fig 5A). Intranasal ORXA 50 μM treatment was effective rescuing the CA-induced IL1β increase in the hippocampus and striatum, hypothalamus and cerebellum (Fig 5A).

TNF-α is a cell signaling cytokine involved in systemic and neuro-inflammation and is one of the cytokines that is involved in the acute phase reaction. Following brain ischemia, expression of TNF-α increases in the ischemic penumbra [47]. Post-CA, TNF-α mRNA expression levels were significantly increased within all brain regions with the maximum response in the hippocampus, striatum, medulla and PFC. Intranasal ORXA 50 μM treatment was able to rescue the CA-induced increases in mRNA TNF-α levels in the hippocampus (Fig 5B).

Nitric oxide is a reactive free radical, which acts as a biologic mediator in several processes, including neurotransmission. iNOS produces large quantities of NO upon stimulation such as by NF-kB and by pro-inflammatory cytokines [48]. In CA+Saline group, iNOS mRNA expression levels increased significantly within all brain regions with the maximum increase observed in the PFC, hippocampus, striatum and hypothalamus. Intranasal ORXA 50 treatment significantly decreased CA-induced iNOS activation in the hippocampus and hypothalamus (Fig 5C).

GFAP is an intermediate filament protein that is expressed mainly by astrocytes. GFAP is involved in many functions including cell communication, the blood brain barrier, and repair after CNS injury. As illustrated in Fig 5D, CA resulted in significant increases in mRNA GFAP levels in the PFC and striatum, while there were no significant increases in other brain regions. Post-CA treatment with ORXA-50 group did not modify mRNA GFAP levels as compared to CA+Saline treated animals (Fig 5D).

CD11b is a cell surface recognition receptor that is used as a marker for microglial activation in the CNS [49]. CD11b is required for the microglia-neuron interaction [50] and is essential for microglial phagocytosis [51]. CA significantly increased CD11b mRNA levels in the PFC, caudal cortex, hippocampus, and striatum. The maximum response was observed in the PFC (Fig 5E). Intranasal ORXA 50 μM treatment prevented CA-induced activation of CD11b in the hippocampus and showed trend inhibiting mRNA CD11b levels in the PFC (Fig 5E).

**Fig 6:**
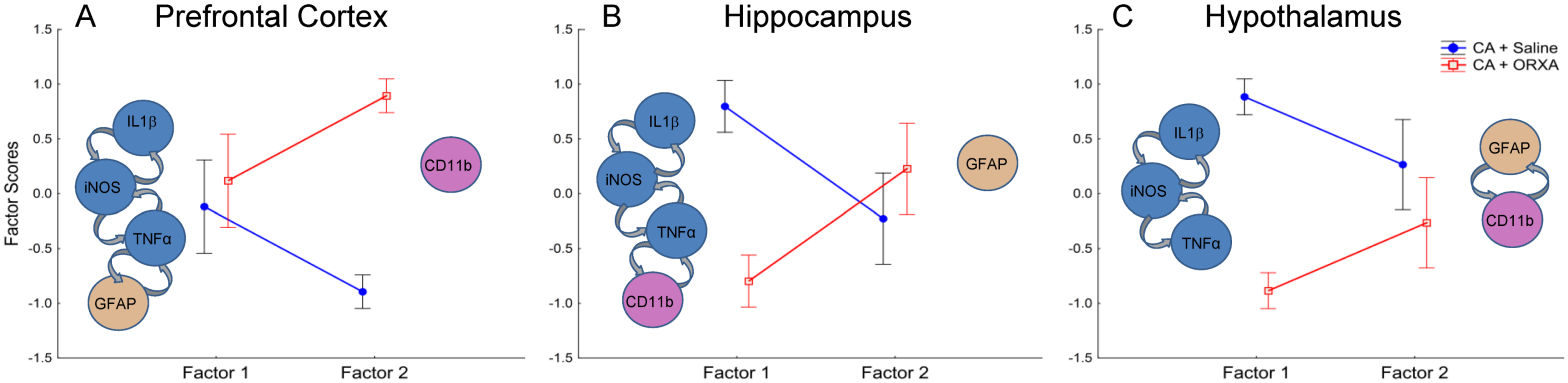
Summary of changes in neuroinflammatory markers induced by CA and ORXA treatment. Raw variables of neuroinflammatory markers were subjected to factor analyses for each of the representative brain structures: (A) the prefrontal cortex, (B) hippocampus, and (C) hypothalamus. Group means ± SEM for Factor scores are shown for CA + Saline and CA + ORXA groups. Inserts in each panel show sets of variables represented by each factor (see S8 Table for Factor loadings). Asterisks indicate significant effect of ORXA, p<0.005, as a result of LSD post-hoc tests (see S9 Table for the ANOVAs of factor scores).

To analyze the extent to which changes in the neuroinflammatory markers were linked with each other, we performed factor analyses (FA) using data from three representative brain structures: the PFC, hippocampus and hypothalamus (Fig 6). For each of the structures, the FA yielded 2 factors that explained more than 85% of variability in the data sets (S8 Table). This indicated that, in each of the brain structures, 5 markers of neuroinflammation can be faithfully represented by two non-correlated integral variables (Factors). In each of the brain structures, measures of cytokines (IL1β, TNFα) and iNOS were co-segregated in one Factor indicating that CA-induced activation of these markers is highly correlated (Fig 6; S8 Table). In the PFC, this Factor also included activation of GFAP, the marker of astroglial filaments that was not activated at this time point in most of other brain structures (Fig 5D). The second Factor in the PFC represented the variability in CD11b that was independent of other measures (Fig 6A; S8 Table). Analysis of factor scores in CA + Saline and CA + ORXA groups revealed that effect of ORXA treatment in the PFC was limited to inhibition of only one marker, CD11b (Fig 6A; S9 Table). Similar analysis in the hippocampus demonstrated that ORXA was more effective than in the PFC and inhibited all neuroinflammatory markers that were activated by CA (Fig 6B; S9 Table). In contrast to the forebrain structures, there was no activation of the structural markers of neuroinflammation in the hypothalamus (GFAP, CD11b) (Fig 5D and 5F). In the latter brain structure, the ORXA treatment was highly effective preventing the concerted activation of IL1β, TNFα, and iNOS after cardiac arrest (Fig 6C; S9 Table).

## Discussion

We used a model of cardiac-arrest induced coma [12,32,38] in rats to assess whether intranasal administration of a pro-arousal drug, ORXA, would promote early recovery. This model is highly relevant to analogous clinical situations in humans, as in all animals full cardiac arrest is followed by a drastic decline in blood pressure, severe acidification and hypercapnia that would result in death unless intensive efforts were made to resuscitate the animal. The return of spontaneous circulation after successful resuscitation does not result in recovery of alertness and marks the onset of a long period of coma, as documented by EEG. To experimentally test the hypothesis that promoting early arousal from coma might have beneficial effects, we used intranasal treatment with ORXA delivered 30 minutes after cardiac arrest. Our study demonstrated that this treatment was effective, triggering neurophysiological signs of arousal as judged by activation of a gamma rhythm on EEG and stabilization of blood pressure. In addition, it ameliorated neuroinflammation and significantly improved behavioral arousal.

These results are consistent with a previous study in which we utilized a similar model, with ORXA delivered by a more invasive icv root [12,30]. As with icv delivery, intranasal ORXA resulted in significant improvement of neurologic deficits documented four hours after cardiac arrest. The neurologic deficit scale used in this study was developed to characterize the complete spectrum of alertness, from coma to full wakefulness [1,32]. Particular benefits of ORXA at the earliest stages of behavioral arousal included improved reactivity to stimuli of different modalities (visual, tactile, acoustic, pain) as well as improved general alertness.

One of the earliest effects of ORXA was observed as early as 30 min after the treatment and consisted of prevention of the decline in the arterial blood pressure that was observed at this stage in the vehicle-treated animals. The improved stability of hemodynamic parameters in the orexin-treated animals might indicate more effective engagement of mechanisms of central regulation of blood pressure. One of the brain structures that is known to mediate the pressor effects of orexins is the rostral ventrolateral medulla [24]. Our data on levels of mRNA orexin receptors documented that the medulla was indeed one of the brain structures with the most rigorous response to intranasal ORXA administration.

In our previous studies, we demonstrated that ORXA, when delivered icv or intra-venously, significantly improved electrophysiological measures of arousal and, in particular, increased the power of the EEG gamma band [12,30,52]. Gamma rhythms on EEG are associated with higher cortical functions such as learning, memory, perception, arousal and consciousness [52–54]. Importantly, recovery of the EEG gamma band is associated with better neurological / behavioral outcomes and survival rate after coma [12,30,52]. In this study, intranasal administration with orexin A was also effective at increasing the power of the EEG gamma band. This is a significant finding, as this route of administration allows for decreasing the dose and avoiding side effects associated with the peripheral treatment. It is also easier to implement in situations of acute care. Maximal orexin-induced changes in the gamma band were detected 40–120 min after the treatment. Of note, ORXA did not increase absolute values of this EEG measure but rather stabilized and prevented a decline in the gamma band observed in the controls. If this decline in EEG gamma power signifies a particularly vulnerable time period during early recovery, timing of treatment with a pro-arousal drug might be critical. Importantly, the timing of intranasal ORXA administration used in this study—30 min after cardiac arrest—was effective in sustaining EEG gamma power over the whole period of gamma band depression.

Improvements in behavioral arousal after intranasal ORXA administration were also associated with significant amelioration of neuroinflammation, as assessed by RT-PCR for multiple cytokines and structural markers of astroglia and microglia. One of the major triggers for ischemia-induced inflammation in the brain is oxidative stress. Cardiac arrest dramatically induced immediate mediators of neuroinflammation such as IL1β, TNFα, and iNOS. mRNA levels of these mediators were highly correlated with each other, consistent with ischemia-induced over-activation of multiple inflammatory pathways [55,56]. Amelioration of the consequences of the global ischemia/reperfusion response could be highly beneficial for preventing secondary neuronal injury due to over-activated inflammatory cascades. Intranasal ORXA administration inhibited the concerted activation of IL1β, TNFα, and iNOS markers in a number of brain structures, including the hippocampus and hypothalamus. Previously, cytokine activation including such key components as TNFα and IL1β have been shown in different models of CA [16,17,57] although the involvement of other cytokines is likely to be affected by type of the model, duration of CA and other procedure-related factors [16,17].

Our data on the beneficial effects of ORXA treatment are in agreement with recent studies showing that orexins have neuroprotective qualities, in part by reducing neuroinflammation [25]. Some studies indicated that orexin receptors and iNOS can be observed in the same brain cells, and that levels of orexins and NO might be co-dependent during waking and sleep [24][26]. In addition, ORXA may act as an immunomodulatory regulator by reducing pro-inflammatory cytokines when tested on microglia [27]. Whether ORXA may exert similar effects on different cell types such as neurons or astrocytes is unclear. CA has been shown to result in cell- and brain-region-specific cytokine production. For example, CA-induced TNFα has being co-localized with neurons and IL1β with astrocytes in striatum [16,17].

Cytokine responses of different brain structures to cardiac arrest were not identical as could be expected from the previous studies in a ventricular fibrillation model of CA [16,17]. In our hands, the hippocampus, frontal cortex and striatum showed the highest response to CA as judged by a fold activation of mRNA levels. Striatum was one of the most reactive brain structures in other CA studies as well [16,17]. The ORXA-induced attenuation of the cytokine activation was also different between brain structures. The hypothalamus was characterized by the highest levels of orexin receptors and was the only brain structure in which ORXA administration increased production of both types of orexin receptors (ORX 1R and ORX 2R). In all other brain structures, ORXA administration resulted in predominant activation of ORX 1R, while inhibiting the ORX 2R pathway. The hypothalamus was effectively protected by ORXA, as induction of IL1β, TNFα, and iNOS decreased. Importantly, in the hypothalamus cardiac arrest did not activate structural markers of neuroinflammation in microglia (CD11b) or astroglia (GFAP). These data suggest that the hypothalamus may be less vulnerable than evolutionarily more modern forebrain structures such as the prefrontal cortex, which responded to the same assault by activation of all measured neuroinflammatory markers. In the latter structure, ORXA administration was not effective at reducing levels of cytokines and had a rather small effect on reducing microglial activation (CD11b). Since intranasal ORXA administration was not as effective in the cortex as in the hypothalamus at dampening neuroinflammation, co-treatment with an anti-inflammatory agent might afford additional benefit, preventing excessive inflammation in brain structures particularly vulnerable to ischemia. It is important to note that changes in mRNA levels detected in this study may not be necessarily translated to changes in protein levels.

The limited effect of ORXA treatment on reducing neuroinflammation in the cortical areas seems somewhat surprising, considering the significant improvement observed in measures of EEG gamma band power. These data indicate that brain structures other than cortex may be responsible for the relative stabilization of the gamma band after ORXA administration. Recent studies documented the origin of gamma band oscillations (GBO) in the proper domain of sub-cortical structures [58]. In particular, a cortical gamma oscillator is dependent on activity of fast-spiking parvalbumin-positive interneurons that elicit gamma oscillations in downstream excitatory neurons [58] [59]. Although cortical gamma rhythms originate in the cortex, orexin can modulate cortical gamma power through the ascending reticular activating system (ARAS)[60]. Recent studies indicate that cortically projecting interneurons in the basal forebrain, the final node of the ARAS [61], are particularly important in stage-dependent control of GBO [62]. Considering that ORXA administration affected a wide range of structures surrounding the basal forebrain (the striatum, hypothalamus and medulla), it is likely that in our study the basal forebrain mediated the effects of ORXA on cortical gamma power as well.

A number of additional questions will need to be addressed in future studies. For example, our model has not incorporated any of the risk factors for global brain ischemia such as aging, vascular disease, or hypertension. Potential protective effects of ORXA may be more difficult to demonstrate in a model that better incorporates those risk factors. We also did not address a question whether it is possible to detect any beneficial effects of ORXA treatment using peripheral biomarkers of neuronal injury (for example, NSE). In addition, it is important to test whether the beneficial effects of ORXA observed at the early time point are translated to biologically significant improvements at later stages of a post-coma recovery. For example, an ICV administration of ORXA has been shown to result in transient improvements at 4hrs post-CA and no differences in NDS or histopathology scores at 72 hrs [12]. If a single-bolus ORXA treatment is indeed not sufficient for longer beneficial effects, repeated dosing should be considered for future efficacy studies. The neuroinflammation after global brain ischemia could be time dependent and also region- and cell type-dependent, collectively presenting multiple therapeutic targets [63–65]. Targeting neuroinflammation may be a complex endeavor that may need to extend past the early reperfusion period. In addition, we could not rule out that the intranasal administration of ORXA may led to some systemic effects that could be responsible, at least in part, for the effects on NDS at 4 hrs. Finally, more mechanistic understanding of the anti-inflammatory effects of ORXA pathways are needed to narrow the inflammatory and therapeutic targets in the brain. More exhaustive studies on precise dosing and timing of delivery would also help in making this therapeutic approach clinically viable to combat consequences of global brain ischemia and coma.

### Conclusions

The results of our study support the idea of beneficial effects of ORXA to facilitate early arousal from coma. On a physiological level, intranasal ORXA administration reduces neuroinflammation and facilitates arousal. First of all, ORXA resulted in stabilization of EEG gamma band power, a measure that is associated with good post-coma neurological prognosis. Further, the EEG measure, IQ, has been shown to correlate with neurological deficits and survival. Secondly, intranasal ORXA administration resulted in significant amelioration of neuroinflammation in multiple brain structures, including the hippocampus and hypothalamus. Our observation showing an improvement in cortical EEG gamma power, despite rather limited effects of ORXA on neuroinflammation in the cortex, seems to indicate that ORXA maintains cortical EEG through activation of subcortical structures. Intranasal delivery of ORXA is a highly clinically translatable route of administration, and EEG based monitoring similarly applicable approach to monitoring and titrating the drug effects. Therefore, our work lays the foundations for undertaking treatment of patients who have experience cardiac arrest and are in subsequent coma.

## Supporting information

S1 Fig (TIF)

S1 Table (DOCX)

S2 Table (DOCX)

S3 Table (DOCX)

S4 Table (DOCX)

S5 Table (DOCX)

S6 Table (DOCX)

S7 Table (DOCX)

S8 Table (DOCX)

S9 Table (DOCX)

S1 File ARRIVE checklist. (PDF)

## Abbreviations

ABG: arterial blood gases
IQ: Information Quantity
NDS: neurologic deficit score
ORXA: Orexin-A
ROSC: return of spontaneous circulation
TH: Therapeutic hypothermia

## References

[1] Geocadin RG, Koenig MA, Jia X, Stevens RD, Peberdy MA. Management of brain injury after resuscitation from cardiac arrest. Neurol Clin. 2008;26: 487–506, ix doi: 10.1016/j.ncl.2008.03.015 18514823 10.1016/j.ncl.2008.03.015 PMC3074242

[2] Stevens RD, Sutter R. Prognosis in severe brain injury. Crit Care Med. 2013;41: 1104–1123. doi: 10.1097/CCM.0b013e318287ee79 23528755 10.1097/CCM.0b013e318287ee79

[3] Peberdy MA, Callaway CW, Neumar RW, Geocadin RG, Zimmerman JL, Donnino M, et al Part 9: post-cardiac arrest care: 2010 American Heart Association Guidelines for Cardiopulmonary Resuscitation and Emergency Cardiovascular Care. Circulation. 2010;122: S768–86. doi: 10.1161/CIRCULATIONAHA.110.971002 20956225 10.1161/CIRCULATIONAHA.110.971002

[4] Gaieski DF, Band RA, Abella BS, Neumar RW, Fuchs BD, Kolansky DM, et al Early goal-directed hemodynamic optimization combined with therapeutic hypothermia in comatose survivors of out-of-hospital cardiac arrest. Resuscitation. 2009;80: 418–424. doi: 10.1016/j.resuscitation.2008.12.015 19217200 10.1016/j.resuscitation.2008.12.015

[5] Nagao K. Therapeutic hypothermia following resuscitation. Curr Opin Crit Care. 2012;18: 239–245. doi: 10.1097/MCC.0b013e3283523f4a 22450742 10.1097/MCC.0b013e3283523f4a

[6] Nielsen N, Wetterslev J, Cronberg T, Erlinge D, Gasche Y, Hassager C, et al Targeted temperature management at 33 C versus 36 C after cardiac arrest. N Engl J Med. 2013;369: 2197–2206. doi: 10.1056/NEJMoa1310519 24237006 10.1056/NEJMoa1310519

[7] Brown RE, Basheer R, McKenna JT, Strecker RE, McCarley RW. Control of sleep and wakefulness. Physiol Rev. 2012;92: 1087–1187. doi: 10.1152/physrev.00032.2011 22811426 10.1152/physrev.00032.2011 PMC3621793

[8] Schmidt MR, Kristiansen SB, Botker HE. Remote ischemic preconditioning: no loss in clinical translation. Circ Res. 2013;113: 1278–1280. doi: 10.1161/CIRCRESAHA.113.302942 24311614 10.1161/CIRCRESAHA.113.302942

[9] España RA, Reis KM, Valentino RJ, Berridge CW. Organization of hypocretin/orexin efferents to locus coeruleus and basal forebrain arousal-related structures. J Comp Neurol. 2005;481: 160–178. doi: 10.1002/cne.20369 15562511 10.1002/cne.20369

[10] Deutschman CS, Raj NR, McGuire EO, Kelz MB. Orexinergic activity modulates altered vital signs and pituitary hormone secretion in experimental sepsis. Crit Care Med. 2013;41: e368–75. doi: 10.1097/CCM.0b013e31828e9843 24105451 10.1097/CCM.0b013e31828e9843 PMC6880745

[11] Sinton CM. Orexin/hypocretin plays a role in the response to physiological disequilibrium. Sleep medicine reviews. 2011;15: 197–207. doi: 10.1016/j.smrv.2010.12.003 21269851 10.1016/j.smrv.2010.12.003

[12] Koenig MA, Jia X, Kang X, Velasquez A, Thakor NV, Geocadin RG. Intraventricular orexin-A improves arousal and early EEG entropy in rats after cardiac arrest. Brain Res. 2009;1255: 153–161. doi: 10.1016/j.brainres.2008.11.102 19111527 10.1016/j.brainres.2008.11.102 PMC4414324

[13] Sakurai T, Amemiya A, Ishii M, Matsuzaki I, Chemelli RM, Tanaka H, et al Orexins and orexin receptors: a family of hypothalamic neuropeptides and G protein-coupled receptors that regulate feeding behavior. Cell. 1998;92: 573–585. 9491897 10.1016/s0092-8674(00)80949-6

[14] Xiang Y, Zhao H, Wang J, Zhang L, Liu A, Chen Y. Inflammatory mechanisms involved in brain injury following cardiac arrest and cardiopulmonary resuscitation. Biomedical reports. 2016;5: 11–17. doi: 10.3892/br.2016.677 27330748 10.3892/br.2016.677 PMC4906809

[15] Patricia B, Benoit SM, Schock S, Plamondon H. CRHR1 exacerbates the glial inflammatory response and alters BDNF/TrkB/pCREB signaling in a rat model of global cerebral ischemia: implications for neuroprotection and cognitive recovery. Prog Neuro-Psychopharmacol Biol Psychiatry. 2017;79: 234–248. 10.1016/j.pnpbp.2017.06.021 28647536

[16] Janata A, Magnet IA, Uray T, Stezoski JP, Janesko-Feldman K, Tisherman SA, et al Regional TNFα mapping in the brain reveals the striatum as a neuroinflammatory target after ventricular fibrillation cardiac arrest in rats. Resuscitation. 2014;85: 694–701. doi: 10.1016/j.resuscitation.2014.01.033 24530249 10.1016/j.resuscitation.2014.01.033 PMC4034695

[17] Drabek T, Wilson CD, Janata A, Stezoski JP, Janesko-Feldman K, Garman RH, et al Unique Brain Region-Dependent Cytokine Signatures After Prolonged Hypothermic Cardiac Arrest in Rats. Therapeutic hypothermia and temperature management. 2015;5: 26–39. doi: 10.1089/ther.2014.0013 25423415 10.1089/ther.2014.0013

[18] Bro-Jeppesen J, Kjaergaard J, Wanscher M, Nielsen N, Friberg H, Bjerre M, et al The inflammatory response after out-of-hospital cardiac arrest is not modified by targeted temperature management at 33 C or 36 C. Resuscitation. 2014;85: 1480–1487. doi: 10.1016/j.resuscitation.2014.08.007 25150183 10.1016/j.resuscitation.2014.08.007

[19] Adrie C, Laurent I, Monchi M, Cariou A, Dhainaou JF, Spaulding C. Postresuscitation disease after cardiac arrest: a sepsis-like syndrome? Curr Opin Crit Care. 2004;10: 208–212. 15166838 10.1097/01.ccx.0000126090.06275.fe

[20] Bro-Jeppesen J, Kjaergaard J, Wanscher M, Nielsen N, Friberg H, Bjerre M, et al Systemic Inflammatory Response and Potential Prognostic Implications After Out-of-Hospital Cardiac Arrest: A Substudy of the Target Temperature Management Trial. Crit Care Med. 2015;43: 1223–1232. doi: 10.1097/CCM.0000000000000937 25756419 10.1097/CCM.0000000000000937

[21] Fries M, Stoppe C, Brücken D, Rossaint R, Kuhlen R. Influence of mild therapeutic hypothermia on the inflammatory response after successful resuscitation from cardiac arrest. J Crit Care. 2009;24: 453–457. doi: 10.1016/j.jcrc.2008.10.012 19327318 10.1016/j.jcrc.2008.10.012

[22] Samborska-Sablik A, Sablik Z, Gaszynski W. The role of the immuno-inflammatory response in patients after cardiac arrest. Archives of Medical Science. 2011;7: 619–626. doi: 10.5114/aoms.2011.24131 22291797 10.5114/aoms.2011.24131 PMC3258769

[23] Callaway CW, Rittenberger JC, Logue ES, McMichael MJ. Hypothermia after cardiac arrest does not alter serum inflammatory markers. Crit Care Med. 2008;36: 2607–2612. doi: 10.1097/CCM.0b013e318184443b 18679114 10.1097/CCM.0b013e318184443b

[24] Xiao F, Jiang M, Du D, Xia C, Wang J, Cao Y, et al Orexin A regulates cardiovascular responses in stress-induced hypertensive rats. Neuropharmacology. 2013;67: 16–24. doi: 10.1016/j.neuropharm.2012.10.021 23147417 10.1016/j.neuropharm.2012.10.021

[25] Xiong X, White RE, Xu L, Yang L, Sun X, Zou B, et al Mitigation of murine focal cerebral ischemia by the hypocretin/orexin system is associated with reduced inflammation. Stroke. 2013;44: 764–770. doi: 10.1161/STROKEAHA.112.681700 23349191 10.1161/STROKEAHA.112.681700 PMC3638929

[26] Kostin A, McGinty D, Szymusiak R, Alam M. Sleep-wake and diurnal modulation of nitric oxide in the perifornical-lateral hypothalamic area: Real-time detection in freely behaving rats. Neuroscience. 2013;254: 275–284. doi: 10.1016/j.neuroscience.2013.09.022 24056193 10.1016/j.neuroscience.2013.09.022

[27] Duffy CM, Yuan C, Wisdorf LE, Billington CJ, Kotz CM, Nixon JP, et al Role of orexin A signaling in dietary palmitic acid-activated microglial cells. Neurosci Lett. 2015;606: 140–144. doi: 10.1016/j.neulet.2015.08.033 26306651 10.1016/j.neulet.2015.08.033 PMC4811357

[28] Kang X, Jia X, Geocadin RG, Thakor NV, Maybhate A. Multiscale entropy analysis of EEG for assessment of post-cardiac arrest neurological recovery under hypothermia in rats. IEEE Transactions on Biomedical Engineering. 2009;56: 1023–1031. doi: 10.1109/TBME.2008.2011917 19174339 10.1109/TBME.2008.2011917 PMC3050512

[29] Jia X, Koenig MA, Venkatraman A, Thakor NV, Geocadin RG. Post-cardiac arrest temperature manipulation alters early EEG bursting in rats. Resuscitation. 2008;78: 367–373. doi: 10.1016/j.resuscitation.2008.04.011 18597914 10.1016/j.resuscitation.2008.04.011 PMC2570264

[30] Akbari Y, Maybhate A, Chen C, Greenwald E, Liang L, Buitrago-Blanco M, et al Orexin-A Improves Arousal from Post—Cardiac Arrest Coma in a Rodent Model. Circulation. 2012;126: A2.

[31] Katz L, Ebmeyer U, Safar P, Radovsky A, Neumar R. Outcome model of asphyxial cardiac arrest in rats. Journal of Cerebral Blood Flow & Metabolism. 1995;15: 1032–1039. 7593335 10.1038/jcbfm.1995.129

[32] Geocadin R, Ghodadra R, Kimura T, Lei H, Sherman D, Hanley D, et al A novel quantitative EEG injury measure of global cerebral ischemia. Clinical Neurophysiology. 2000;111: 1779–1787. 11018492 10.1016/s1388-2457(00)00379-5

[33] Benson DM, O’Neil B, Kakish E, Erpelding J, Alousi S, Mason R, et al Open-chest CPR improves survival and neurologic outcome following cardiac arrest. Resuscitation. 2005;64: 209–217. doi: 10.1016/j.resuscitation.2003.03.001 15680532 10.1016/j.resuscitation.2003.03.001

[34] Bendel S, Springe D, Pereira A, Grandgirard D, Leib SL, Putzu A, et al Do different anesthesia regimes affect hippocampal apoptosis and neurologic deficits in a rodent cardiac arrest model? BMC anesthesiology. 2015;15: 2 doi: 10.1186/1471-2253-15-2 25972075 10.1186/1471-2253-15-2 PMC4429377

[35] Zuercher P, Springe D, Grandgirard D, Leib SL, Grossholz M, Jakob S, et al A randomized trial of the effects of the noble gases helium and argon on neuroprotection in a rodent cardiac arrest model. BMC neurology. 2016;16: 43 doi: 10.1186/s12883-016-0565-8 27044425 10.1186/s12883-016-0565-8 PMC4820914

[36] Jia X, Koenig MA, Shin H, Zhen G, Pardo CA, Hanley DF, et al Improving neurological outcomes post-cardiac arrest in a rat model: immediate hypothermia and quantitative EEG monitoring. Resuscitation. 2008;76: 431–442. doi: 10.1016/j.resuscitation.2007.08.014 17936492 10.1016/j.resuscitation.2007.08.014 PMC2323440

[37] Geocadin RG, Muthuswamy J, Sherman DL, Thakor NV, Hanley DF. Early electrophysiological and histologic changes after global cerebral ischemia in rats. Mov Disord. 2000;15 Suppl 1: 14–21. 10755267 10.1002/mds.870150704

[38] Jia X, Koenig MA, Shin H, Zhen G, Yamashita S, Thakor NV, et al Quantitative EEG and neurological recovery with therapeutic hypothermia after asphyxial cardiac arrest in rats. Brain Res. 2006;1111: 166–175. doi: 10.1016/j.brainres.2006.04.121 16919609 10.1016/j.brainres.2006.04.121 PMC3074257

[39] Sherman D, Hinich M, Thakor N. The higher order statistics of energy operators with application to neurological signals. 1998: 561–564.

[40] Claassen J, Velasquez A, Meyers E, Witsch J, Falo CM, Park S, et al Bedside quantitative EEG improves assessment of consciousness in comatose subarachnoid hemorrhage patients. Ann Neurol. 2016;80:541–553. doi: 10.1002/ana.24752 27472071 10.1002/ana.24752 PMC5042849

[41] Zhang JM, An J. Cytokines, inflammation, and pain. Int Anesthesiol Clin. 2007;45: 27–37. doi: 10.1097/AIA.0b013e318034194e 17426506 10.1097/AIA.0b013e318034194e PMC2785020

[42] Livak KJ, Schmittgen TD. Analysis of relative gene expression data using real-time quantitative PCR and the 2− ΔΔCT method. Methods. 2001;25: 402–408. doi: 10.1006/meth.2001.1262 11846609 10.1006/meth.2001.1262

[43] Deng R, Koenig MA, Young LM, Jia X. Early quantitative gamma-band EEG marker is associated with outcomes after cardiac arrest and targeted temperature management. Neurocritical care. 2015;23: 262–273. doi: 10.1007/s12028-015-0157-2 26130405 10.1007/s12028-015-0157-2 PMC4560606

[44] Nambu T, Sakurai T, Mizukami K, Hosoya Y, Yanagisawa M, Goto K. Distribution of orexin neurons in the adult rat brain. Brain Res. 1999;827: 243–260. 10320718 10.1016/s0006-8993(99)01336-0

[45] Chaudhry H, Zhou J, Zhong Y, Ali MM, McGuire F, Nagarkatti PS, et al Role of cytokines as a double-edged sword in sepsis. In Vivo. 2013;27: 669–684. 24292568 PMC4378830

[46] Kim Y, Hwang S, Oh E, Oh S, Han I. IL-1β, an immediate early protein secreted by activated microglia, induces iNOS/NO in C6 astrocytoma cells through p38 MAPK and NF-κB pathways. J Neurosci Res. 2006;84: 1037–1046. doi: 10.1002/jnr.21011 16881054 10.1002/jnr.21011

[47] Lambertsen KL, Biber K, Finsen B. Inflammatory cytokines in experimental and human stroke. Journal of Cerebral Blood Flow & Metabolism. 2012;32: 1677–1698. 22739623 10.1038/jcbfm.2012.88 PMC3434626

[48] Sprague AH, Khalil RA. Inflammatory cytokines in vascular dysfunction and vascular disease. Biochem Pharmacol. 2009;78: 539–552. doi: 10.1016/j.bcp.2009.04.029 19413999 10.1016/j.bcp.2009.04.029 PMC2730638

[49] Linnartz B, Neumann H. Microglial activatory (immunoreceptor tyrosine-based activation motif)-and inhibitory (immunoreceptor tyrosine-based inhibition motif)-signaling receptors for recognition of the neuronal glycocalyx. Glia. 2013;61: 37–46. doi: 10.1002/glia.22359 22615186 10.1002/glia.22359

[50] Wakselman S, Bechade C, Roumier A, Bernard D, Triller A, Bessis A. Developmental neuronal death in hippocampus requires the microglial CD11b integrin and DAP12 immunoreceptor. J Neurosci. 2008;28: 8138–8143. doi: 10.1523/JNEUROSCI.1006-08.2008 18685038 10.1523/JNEUROSCI.1006-08.2008 PMC6670768

[51] Linnartz B, Kopatz J, Tenner AJ, Neumann H. Sialic acid on the neuronal glycocalyx prevents complement C1 binding and complement receptor-3-mediated removal by microglia. J Neurosci. 2012;32: 946–952. doi: 10.1523/JNEUROSCI.3830-11.2012 22262892 10.1523/JNEUROSCI.3830-11.2012 PMC4037907

[52] Cantero JL, Atienza M, Madsen JR, Stickgold R. Gamma EEG dynamics in neocortex and hippocampus during human wakefulness and sleep. Neuroimage. 2004;22: 1271–1280. doi: 10.1016/j.neuroimage.2004.03.014 15219599 10.1016/j.neuroimage.2004.03.014

[53] Uhlhaas P, Pipa G, Lima B, Melloni L, Neuenschwander S, Nikolić D, et al Neural synchrony in cortical networks: history, concept and current status. Frontiers in integrative neuroscience. 2009;3: 17 doi: 10.3389/neuro.07.017.2009 19668703 10.3389/neuro.07.017.2009 PMC2723047

[54] Uhlhaas PJ, Singer W. Abnormal neural oscillations and synchrony in schizophrenia. Nature reviews neuroscience. 2010;11: 100–113. doi: 10.1038/nrn2774 20087360 10.1038/nrn2774

[55] Wang Y, Hsu Y, Wu H, Lee G, Yang Y, Wu J, et al Endothelium-Derived 5-Methoxytryptophan Is a Circulating Anti-inflammatory Molecule that Blocks Systemic Inflammation. Circ Res. 2016;119:222–236. doi: 10.1161/CIRCRESAHA.116.308559 27151398 10.1161/CIRCRESAHA.116.308559

[56] Marchant DJ, Boyd JH, Lin DC, Granville DJ, Garmaroudi FS, McManus BM. Inflammation in myocardial diseases. Circ Res. 2012;110: 126–144. doi: 10.1161/CIRCRESAHA.111.243170 22223210 10.1161/CIRCRESAHA.111.243170

[57] Liu T, Clark RK, McDonnell PC, Young PR, White RF, Barone FC, et al Tumor necrosis factor-alpha expression in ischemic neurons. Stroke. 1994;25: 1481–1488. 8023366 10.1161/01.str.25.7.1481

[58] Sohal VS, Zhang F, Yizhar O, Deisseroth K. Parvalbumin neurons and gamma rhythms enhance cortical circuit performance. Nature. 2009;459: 698–702. doi: 10.1038/nature07991 19396159 10.1038/nature07991 PMC3969859

[59] Cardin JA, Carlén M, Meletis K, Knoblich U, Zhang F, Deisseroth K, et al Driving fast-spiking cells induces gamma rhythm and controls sensory responses. Nature. 2009;459: 663–667. doi: 10.1038/nature08002 19396156 10.1038/nature08002 PMC3655711

[60] Munk MH, Roelfsema PR, Konig P, Engel AK, Singer W. Role of reticular activation in the modulation of intracortical synchronization. Science. 1996;272: 271–274. 8602512 10.1126/science.272.5259.271

[61] Jones BE, Hassani OK. The role of Hcrt/Orx and MCH neurons in sleep-wake state regulation. Sleep. 2013;36: 1769–1772. doi: 10.5665/sleep.3188 24293746 10.5665/sleep.3188 PMC3825421

[62] Kim T, Thankachan S, McKenna JT, McNally JM, Yang C, Choi JH, et al Cortically projecting basal forebrain parvalbumin neurons regulate cortical gamma band oscillations. Proc Natl Acad Sci U S A. 2015;112: 3535–3540. doi: 10.1073/pnas.1413625112 25733878 10.1073/pnas.1413625112 PMC4371918

[63] Saito K, Suyama K, Nishida K, Sei Y, Basile AS. Early increases in TNF-α, IL-6 and IL-1β levels following transient cerebral ischemia in gerbil brain. Neurosci Lett. 1996;206: 149–152. 8710173 10.1016/s0304-3940(96)12460-5

[64] Murakami Y, Saito K, Hara A, Zhu Y, Sudo K, Niwa M, et al Increases in tumor necrosis factor-α following transient global cerebral ischemia do not contribute to neuron death in mouse hippocampus. J Neurochem. 2005;93: 1616–1622. doi: 10.1111/j.1471-4159.2005.03163.x 15935078 10.1111/j.1471-4159.2005.03163.x

[65] Yasuda Y, Shimoda T, Uno K, Tateishi N, Furuya S, Tsuchihashi Y, et al Temporal and sequential changes of glial cells and cytokine expression during neuronal degeneration after transient global ischemia in rats. Journal of neuroinflammation. 2011;8: 70 doi: 10.1186/1742-2094-8-70 21696573 10.1186/1742-2094-8-70 PMC3131233

