# Supplementary figures and images for: Intranasal post-cardiac arrest treatment with orexin-A facilitates arousal from coma and ameliorates neuroinflammation
Source: PLoS One. 2017 Sep 28;12(9):e0182707. doi: 10.1371/journal.pone.0182707 (PMC5619710; doi:10.1371/journal.pone.0182707)

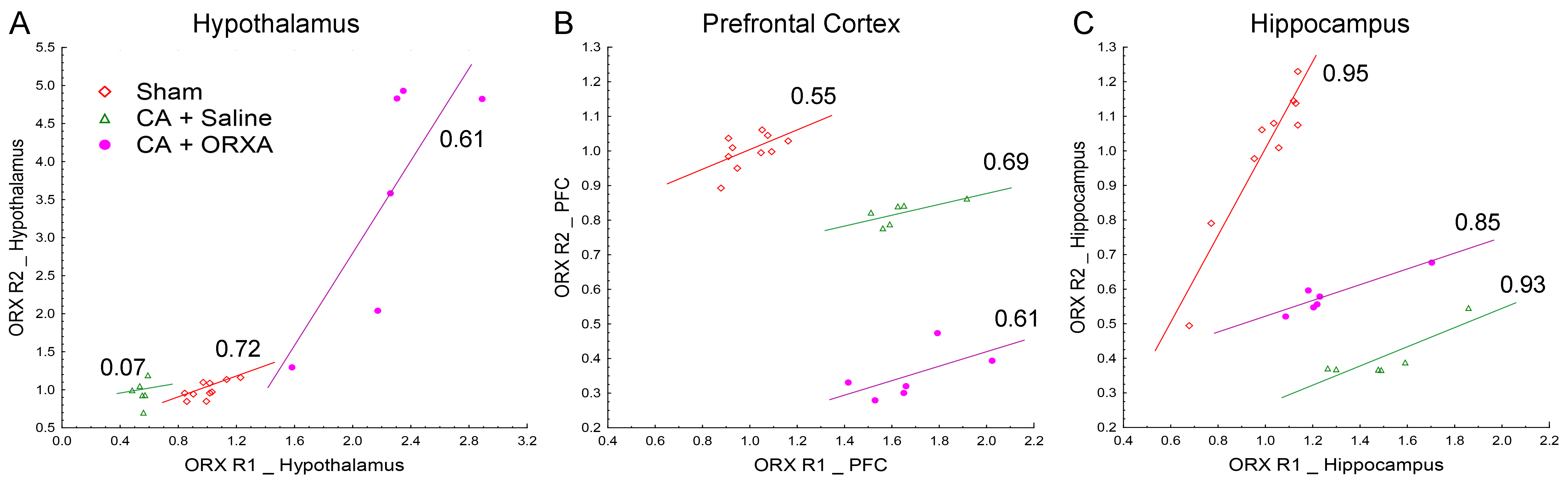

Supplement: S1 Fig — (TIF) [file pone.0182707.s001.tif]

**Table S1: Neurologic Deficit Scale Score (NDS score)**

**
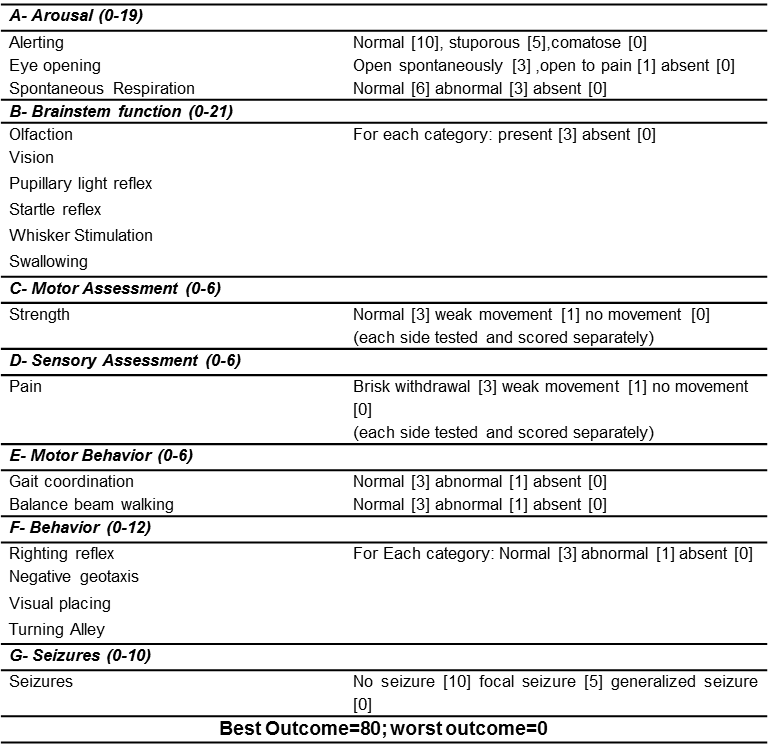
**

Supplement: S1 Table — (DOCX) [file pone.0182707.s002.docx]
