# Supplementary material for: Intranasal post-cardiac arrest treatment with orexin-A facilitates arousal from coma and ameliorates neuroinflammation
Source: PLoS One. 2017 Sep 28;12(9):e0182707. doi: 10.1371/journal.pone.0182707 (PMC5619710; doi:10.1371/journal.pone.0182707)
Supplement: S2 Table — (DOCX) [file pone.0182707.s003.docx]

**Table S2: TaqMan gene expression assays ID for primers.**

| **Target gene** | **Assay ID from Applied Biosystems** |
| --- | --- |
| **IL1β** | **Rn00580432_m1** |
| **TNF-α** | **Rn01525859_g1** |
| **iNOS** | **Rn99999069_mH** |
| **GFAP** | **Rn01253033_m1** |
| **Cd11b** | **Rn00709342_m1** |
| **ORX1 R** | **Rn00565032_m1** |
| **ORX2 R** | **Rn00565155_m1** |
