# Supplementary material for: Intranasal post-cardiac arrest treatment with orexin-A facilitates arousal from coma and ameliorates neuroinflammation
Source: PLoS One. 2017 Sep 28;12(9):e0182707. doi: 10.1371/journal.pone.0182707 (PMC5619710; doi:10.1371/journal.pone.0182707)
Supplement: S3 Table — (DOCX) [file pone.0182707.s004.docx]

**Table S3: Statistics for ANOVA analyses of NDS total scores and subscores.**

| **NDS scores** | **Group** | **Subscores** | **Group x Subscore**  **interaction** |
| --- | --- | --- | --- |
| One-way ANOVA for total NDS  (Fig. 2A) | Df=2,26  F=3.890, p<0.034 | NA | NA |
| Two-way ANOVA  for subscores  (Fig.2C) | Df=2,26  F=4.000, p<0.031 | Df=5,130  F=112.27, p<0.0000 | Df=10,130  F=4.14, p<0.0001 |
