# Supplementary material for: Intranasal post-cardiac arrest treatment with orexin-A facilitates arousal from coma and ameliorates neuroinflammation
Source: PLoS One. 2017 Sep 28;12(9):e0182707. doi: 10.1371/journal.pone.0182707 (PMC5619710; doi:10.1371/journal.pone.0182707)
Supplement: S4 Table — (DOCX) [file pone.0182707.s005.docx]

**Table S4: Spearman Rank Order Correlations between individual NDS scores at 4 hrs post-CA. Scores that had no variability at this time point are not included in the analysis. Marked correlations are significant at p <0.010.**

**
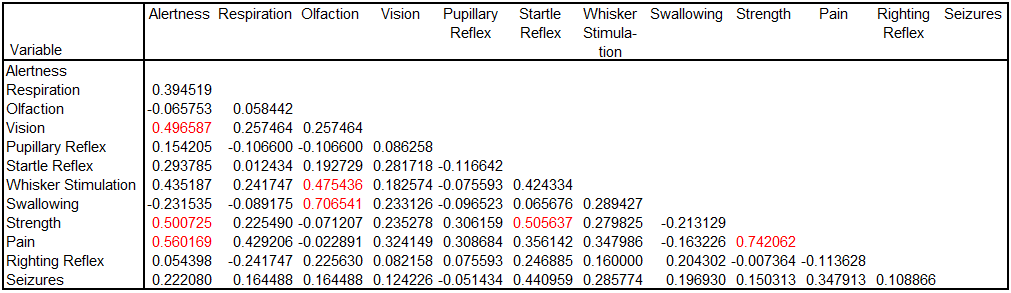
**
