# Supplementary material for: Intranasal post-cardiac arrest treatment with orexin-A facilitates arousal from coma and ameliorates neuroinflammation
Source: PLoS One. 2017 Sep 28;12(9):e0182707. doi: 10.1371/journal.pone.0182707 (PMC5619710; doi:10.1371/journal.pone.0182707)
Supplement: S5 Table — (DOCX) [file pone.0182707.s006.docx]

**Table S5: Results of statistical analyses for EEG gamma power derived from electrode B (see Methods).**

Two-way repeated measures ANOVA was used with main effect of group (CA+Saline: n=14, CA+ORXA50, n=6) and Time Blocks (5 min) as repeated measures. Group means±SEM are presented in Fig. 3.

| **EEG γ power** | **Group** | **Time Block** | **Group x Time Interaction** |
| --- | --- | --- | --- |
| Baseline γ fraction (BL on Fig. 3A-B) | Df=1,18  F=0.21, p>0.65 | Df=1,18  F=1.07  p>0.31 | Df=1,18  F=0.01  p>0.91 |
| γ fraction in the 1^st^ 30 min after CA  (ROCS on Fig. 3A,C) | Df=1,18  F=0.01, p>0.81 | Df=5,90  F=3.12  p<0.012 | Df=5,90  F=1.45  p>0.21 |
| γ fraction after ORXA50  (Drug on Fig. 3A, D) | Df=1,18  F=6.37, p<0.021 | Df=41,738  F=5.88  P<0.0001 | Df=41,738  F=0.57  p>0.98 |
