# Supplementary material for: Intranasal post-cardiac arrest treatment with orexin-A facilitates arousal from coma and ameliorates neuroinflammation
Source: PLoS One. 2017 Sep 28;12(9):e0182707. doi: 10.1371/journal.pone.0182707 (PMC5619710; doi:10.1371/journal.pone.0182707)
Supplement: S6 Table — (DOCX) [file pone.0182707.s007.docx]

**Table S6:** Within-group linear correlations between mRNA levels of ORX R1 and ORX R2 in different brain regions. In the CA+Saline and CA+ORX groups, brains samples were taken 4 hours after the return of spontaneous circulation (ROSC). N indicates number of rats per group. ND, not determined. P level of significance corrected by number of comparisons (Bonferroni correction) is 0.0063.

| **Variable** | **Sham**  **n=10** | **CA+Saline**  **n=6** | **CA+ORXA**  **n=6** |
| --- | --- | --- | --- |
| **PF Cortex** | 0.5535 | 0.6883 | 0.6129 |
|  | p=0.097 | p=0.131 | p=0.196 |
| **Som. Cortex** | 0.6671 | 0.1078 | 0.1747 |
|  | p=0.035 | p=0.839 | p=0.741 |
| **Hippocampus** | 0.9464 | 0.8510 | 0.9255 |
|  | p=0.000 | p=0.032 | p=0.008 |
| **Striatum** | 0.7544 | 0.8739 | 0.3546 |
|  | p=0.012 | p=0.023 | p=0.490 |
| **Hypothalamus** | 0.7209 | 0.0737 | 0.7990 |
|  | p=0.019 | p=0.890 | p=0.057 |
| **Medulla** | 0.5878 | 0.0242 | ND |
|  | p=0.074 | p=0.964 |  |
| **ROB** | 0.8727 | 0.3726 | 0.7307 |
|  | p=.001 | p=0.467 | p=0.099 |
| **Cerebellum** | 0.5247 | 0.3643 | 0.4306 |
|  | p=0.119 | p=0.478 | p=0.394 |
