# Supplementary material for: Intranasal post-cardiac arrest treatment with orexin-A facilitates arousal from coma and ameliorates neuroinflammation
Source: PLoS One. 2017 Sep 28;12(9):e0182707. doi: 10.1371/journal.pone.0182707 (PMC5619710; doi:10.1371/journal.pone.0182707)
Supplement: S7 Table — (DOCX) [file pone.0182707.s008.docx]

**Table S7:** Results of statistical analyses for mRNA levels of cytokines and ORX R mRNA levels in different brain structures.

Two-way repeated measures ANOVA was used with main effect of group (Sham, n=10; CA+Saline, n=6; CA+ORXA 50, n=6) and different brain regions as repeated measures. Group means±SEM are presented in Fig. 4-5. LSD post-hoc test was applied to significant Group x Structure interactions to test for differences in particular set of means (shown in Fig. 4). P level of significance was corrected by the number of comparisons within each ANOVA (Bonferroni correction, Pcor < 0.0063).

| **Markers** | **Group** | **Brain Structure** | **Group x Structure Interaction** |
| --- | --- | --- | --- |
| IL1β | Df=2,133  F=441.09, p<0.0001 | Df=7,133  F=26.25  P<0.0001 | Df=14,133  F=40.21  p<0.0001 |
| iNOS | Df=2,133  F=394.36 p<0.0001 | Df=7,133  F=51.276  P<0.0001 | Df=14,133  F=29.61  p<0.0001 |
| TNF-α | Df=2,133  F=910.93 p<0.0001 | Df=7.0,133  F=191.65  P<0.0001 | Df=14,133  F=83.62  P<0.0001 |
| GFAP | Df=2,133  F=16.489  P<0.0001 | Df=7,133  F=31.709  P<0.0001 | Df=14,133  F=10.078  P<0.0001 |
| Cd11b | Df=2,133  F=345.34  P<0.0001 | Df=7,133  F=140.97  P<0.00001 | Df=14,133  F=71.98  P<0.0001 |
| ORX1R | Df=2,133  F=317.67  P<0.0001 | Df=7,133  F=167.13  P<0.0001 | Df=14,133  F=56.58  P<0.0001 |
| ORX2R | Df=2,133  F=7.976  P=0.0031 | Df=7,133  F=35.199  P<0.0001 | Df=14,133  F=29.914  P<0.0001 |
