# Supplementary material for: Intranasal post-cardiac arrest treatment with orexin-A facilitates arousal from coma and ameliorates neuroinflammation
Source: PLoS One. 2017 Sep 28;12(9):e0182707. doi: 10.1371/journal.pone.0182707 (PMC5619710; doi:10.1371/journal.pone.0182707)

**Table S8:** Results of factor analyses for mRNA levels of neuroinflammatory markers in three representative brain structures. Data from CA + Saline and CA + ORXA groups were included in the analyses. Factor loadings for two main factors (eigenvalue >1) were subjected to a Varimax normalized rotation to facilitate interpretation while preserving orthogonality of the factors. Factor loadings higher than 0.80 are highlighted in red. *Expl.Var* – explained variability; *Prp.Totl* – proportion of total variability explained by a factor.


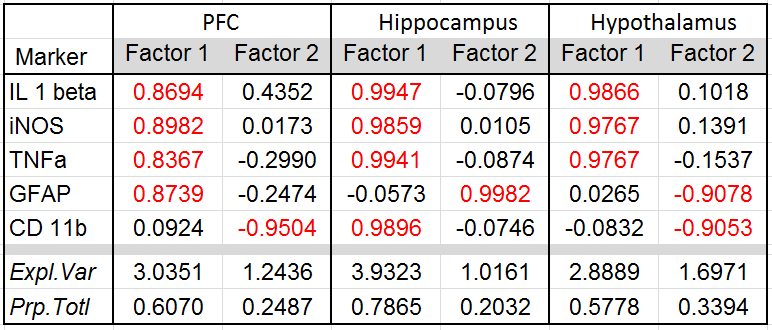

Supplement: S8 Table — (DOCX) [file pone.0182707.s009.docx]
