# Supplementary material for: Intranasal post-cardiac arrest treatment with orexin-A facilitates arousal from coma and ameliorates neuroinflammation
Source: PLoS One. 2017 Sep 28;12(9):e0182707. doi: 10.1371/journal.pone.0182707 (PMC5619710; doi:10.1371/journal.pone.0182707)
Supplement: S9 Table — (DOCX) [file pone.0182707.s010.docx]

**Table S9:** Structure and results of ANOVAs for Factor scores (see Table S8 for Factors’ loadings).

| **Brain Structure** | **Group** | **Repeated Measures**  **(Factors)** | **Group x Factor Interaction** |
| --- | --- | --- | --- |
| PFC | Df=1,10  F=12.65, p<0.0052 | Df=1,110  F=0.00,  p<1.000 | Df=1,10  F=4.89,  p<0.05 |
| Hippocampus | Df=1,10  F=2.15, p>0.18 | Df=1,110  F=0.00  p<1.000 | Df=1,10  F=13.41  p<0.0043 |
| Hypothalamus | Df=1,10  F=25.83, p<0.0005 | Df=1,110  F=0.00  p<1.000 | Df=1,10  F=2.65  p>0.13 |
